# Care performed by family caregivers of children submitted to hematopoietic stem cell transplantation*

**DOI:** 10.1590/1518-8345.2298-3120

**Published:** 2019-01-14

**Authors:** Ingrid Meireles Gomes, Maria Ribeiro Lacerda, Ana Paula Hermann, Jéssica Alline Pereira Rodrigues, Débora Cristina Paes Zatoni, Luana Tonin

**Affiliations:** 1 Universidade Federal do Paraná, Hospital de Clínicas, Curitiba, PR, Brazil.; 2 Universidade Federal do Paraná, Departamento de Enfermagem e Terapia Ocupacional, Curitiba, PR, Brazil.

**Keywords:** Hematopoietic Stem Cell Transplantation, Caregivers, Family, Child Care, Bone Marrow Transplantation, Housing, Transplante de Células-Tronco Hematopoiéticas, Cuidadores, Família, Cuidado da Criança, Transplante de Medula Óssea, Habitação, Trasplante de Células Madre Hematopoyéticas, Cuidadores, Familia, Cuidado del Niño, Trasplante de Médula Ósea, Vivienda

## Abstract

**Objective:**

to know the care provided by family caregivers of children submitted to hematopoietic stem cell transplantation.

**Method:**

the Grounded Theory was used as methodology. The study comprised four sample groups, comprising 36 caregivers. Data were collected by semi-structured interviews and analyzed according to the coding proposed by Strauss and Corbin in three phases: open, axial and selective.

**Results:**

eight propositions were identified for the care provided to the child in the researched context, namely administering medications; attention to cleaning issues; care with water and food intake; care with the body; experiencing protective isolation; addressing the child’s need for emotional support; addressing the child’s self-care; and facing complications.

**Conclusion:**

the different aspects in which the caregiver acts in the care of the child were understood. Such care equips the health team to elaborate measures for guidance and preparation of home care that are effective and directed to the needs of the patient and their family. The understanding of the care that they accomplish enables the caregiver a greater understanding of their role, as well as of the decisions they will make by their being under treatment.

## RESUMO

**Objetivo:**

conhecer os cuidados realizados pelo cuidador familiar da criança em pós-transplante de células-tronco hematopoiéticas.

**Método:**

utilizou-se a *Grounded Theory* como metodologia; compreendendo quatro grupos amostrais, perfazendo 36 cuidadores; com dados coletados por entrevistas semiestruturadas. Os dados foram analisados segundo a codificação proposta por Strauss e Corbin, em três fases: aberta, axial e seletiva.

**Resultados:**

foram identificadas oito proposições para os cuidados realizados com criança no contexto pesquisado: administrar medicações; atentar às questões de limpeza; cuidados com ingesta hídrica e alimentar; cuidados com o corpo; experienciar o isolamento protetor; trabalhar a necessidade de apoio emocional da criança; abordar o autocuidado da criança; e encarar complicações.

**Conclusão:**

compreenderam-se as diferentes vertentes em que o cuidador atua no cuidado com a criança. Esses cuidados instrumentalizam a equipe de saúde ao nortear a elaboração de medidas de orientação e preparo para o cuidado domiciliar que sejam efetivas e direcionadas às necessidades do paciente e da família. A compreensão dos cuidados que realiza viabiliza ao cuidador maior entendimento do seu papel, bem como das decisões que tomará pelo seu ente em tratamento.

## RESUMEN

**Objetivo:**

conocer los cuidados realizados por el cuidador familiar del niño después del trasplante de células madre hematopoyéticas.

**Método:**

se utilizó la *Grounded Theory* como metodología; comprendió cuatro grupos de muestreo, totalizando 36 cuidadores; utilizó datos recogidos por medio de entrevistas semiestructuradas, los datos fueron analizados según la codificación propuesta por Straus y Corbin, en tres fases: abierta, axial y selectiva.

**Resultados:**

fueron identificadas ocho proposiciones para los cuidados realizados al niño en el siguiente contexto investigado: administrar medicaciones; prestar atención a las cuestiones de limpieza; cuidados con ingesta hídrica y alimentar; cuidados con el cuerpo; experimentar el aislamiento protector; trabajar la necesidad de dar apoyo emocional al niño; abordar el autocuidado del niño; y, encarar las complicaciones.

**Conclusión:**

fueron comprendidas las diferentes vertientes en las cuales el cuidador actúa en el cuidado al niño. Esos cuidados instrumentalizan al equipo de salud para orientar la elaboración de medidas de orientación y preparación, para el cuidado domiciliar, que sean efectivas y dirigidas a las necesidades del paciente y su familia. La comprensión de los cuidados que realiza viabiliza al cuidador obtener mayor entendimiento de su papel, así como de las decisiones que tomará auxiliar a su familiar en tratamiento.

## Introduction

In a variety of illnesses, hematopoietic stem cell transplantation (HSCT) becomes the most viable treatment option, such as malignant, hereditary, immunological, metabolic and oncological hematologic diseases^1^. This procedure is in full expansion and reaches more and more positive results, mainly due to technological advances, which represent its potentiality, but may also be its weakness^2^.

Although this procedure is still evolving, much has been studied and developed in relation to quantitative, operational and structural questions, and subjective issues still need to be better discussed and deepened, since, even in the face of this favorable evolution, the risk of mortality and physical and psychosocial morbidity remains related to the procedure^3^. Besides the patient, this procedure also has a physical, emotional and psychological impact on the family caregiver^1^ ^,^ ^3^ who, although it is not the object of transplantation, faces repercussions in their private life, as well as in their social group, especially when it comes to family caregivers of children who are submitted to HSCT, in view of the dependence inherent of childhood.

The HSCT is a long treatment, composed of distinct phases that present two marked differences: the place where care occurs and the main responsible for these phases of care. During hospitalization, the direct care is performed by the health team in a hospital environment; after the discharge, the home represents the main environment of care and it is the family caregiver who is responsible for the decisions, remains with the patient most of the time and takes care of most of the care actions, even during the outpatient treatment after discharge from hospital.

Even if the family caregiver should not be held responsible for the result of the transplantation, since the success thereof depends, among other things, on inherent aspects of the disease itself, tolerance and response to treatment, a large number of care actions are necessary during the time the patient remains at home, and the family caregiver will be the one responsible for carrying them out. The relevance of the family caregiver is therefore unquestionable^4^ ^-^ ^5^, but not exclusive, for the success of HSCT.

When it comes to child care, this issue is even more prominent, since children are dependent beings because of their natural condition as an infant and because the caregiver previously presents a referential for the care of the child, which allows better understanding and action in situations that may emerge, such as illness, as well as in the use of the potentialities and limitations of the child in the face of the experience faced^6^.

In spite of the quantitative approach in research on HSCT, the literature on the caregiver in this scenario has been growing in recent years and strengthening its importance for transplantation. These surveys are mainly associated with issues about their quality of life, overload and impact or influences that being a caregiver entails in their private or social life^1^ ^,^ ^3^ ^,^ ^5^. However, although the care that is needed in the post-HSCT period can be identified^7^, there are no studies aimed at describing and understanding the care that these subjects actually perform and this work is dedicated to this knowledge gap.

We tried to answer the question: What are the care that the family caregiver of a child submitted to hematopoietic stem cell transplantation performs when the care happens to occur within the home environment? To answer this question, the objective is to know the care taken by the family caregiver of the child submitted to hematopoietic stem cell transplantation.

## Method

The present study used the Grounded Theory as methodology and the symbolic interactionism as a theoretical reference. The Grounded Theory allows the creation of a theory based on data from a given social experience through the identification, development and relationship between concepts, which is given through a thorough comparative analysis, strictly related to the collection of data in a process called circularity of data^8^.

Symbolic interactionism, in turn, is a perspective of empirical social science that seeks to understand human behavior by analyzing the actions, interactions and reactions of individuals through the construction of meanings and interpretation of the identified symbols^9^.

The study was carried out in a Bone Marrow Transplant Service of a large hospital in the southern region of Brazil, a center of excellence and worldwide reference in the treatment of some diseases by HSCT; in a transitional support house (CTA in Portuguese) that offers lodging and services to families of children and adolescents with cancer; and, at the home of some family caregivers.

Participants totaled 36 caregivers of children submitted to HSCT, who fit into four sample groups. The first sample group consisted of 10 caregivers living in CTA during outpatient follow-up at the time of the interview. The decision by the first sample group was intentional, since most of the children attended at the service where the study was conducted come from other cities and remain in transitional homes during the treatment. From this first group, each concluded sample group revealed gaps in the knowledge of the phenomenon, which led to the elaboration of hypotheses, which directed the selection of the members of the subsequent sample group and so on, as proposed in the grounded theory.

The data from the first sample group led to the hypothesis that the experience of home care in CTA was complemented by home care after leaving this environment and by returning to daily life, raising the question whether caring in CTA and in private homes would have similar characteristics or not. Therefore, the second group was composed of seven caregivers who, during the outpatient follow-up, remained in temporary, supportive or private homes, but at the time of the interview they were already in their home address.

Data from the first two groups led to the hypothesis that the place where care is performed after hospital discharge influences the meaning of the actions and interactions that occur in home care. To elucidate this hypothesis, the third sample group was composed of 11 caregivers who returned straight to their own home after discharge.

The data from the three previous groups raised the hypothesis that facing more than one transplantation modifies the experience of home care after HSCT, which gave rise to the fourth sample group, composed of eight caregivers of children who had experienced more than one HSCT, regardless of the place of living after the HSCT. The number of participants who composed each sample group was decided according to the emergence or not of new information, categories or concepts, in the same way as it occurred for the closing of data collection, when the theoretical saturation occurred, also characteristic of grounded theory.

Data collection took place between 2013 and 2016, and was performed according to the consultations scheduled in the service where the study occurred through semi-structured interviews with open questions. A pilot interview was conducted prior to the beginning of the data collection, and data from this interview were not considered for the research results. This pilot interview made it possible to adapt the instrument, which was also modified at each new interview, based on the obtained information and on those that were still sought, as proposed by the methodology. To perform the data collection, external interviewers were used to avoid biases and ethical dilemmas related to this choice. The researchers followed an action plan to develop this proposal, composed of the following steps: feasibility; selection; negotiation; training; and follow-up. The description of this action plan and of this experience with an external interviewer is found in an article published in 2016^10^. In the presentation of the results, the speeches of the participants will be followed by the acronym SG, representing the sample group to which they belong, and the letter P, identifying the participant within the sample group.

Some analytical tools were used, such as the Nvivo10^®^ support software for storing and organizing collected data, data analysis and results presentation; the writing of four-type memoranda (methodological or emerging, reflective or observational, conceptual or theoretical and explanatory of model description^11^); and drawing diagrams. Data analysis was performed using the coding method proposed by Strauss and Corbin^12^, subdivided into three stages: open, axial and selective coding. The outsourced external interviewer did not participate in the analysis of the data, which was done by the main investigator after each interview and before the next one. The discussion with the other researchers was held through the shared use of Nvivo10^®^ and in face-to-face meetings. There were no disagreements between the authors, since the methodology presupposes that the phenomenon is based on the data and this was crucial for the construction of the theory in this study.

The phenomenon under study proved to be a basic social process, so we considered the use of the coding paradigm^12^ to explain the organization of this phenomenon, which was then structured into five components: causes, intervening conditions, context, strategies and consequences. Then, the organized data allowed the construction of a substantive theory entitled “Experience of family home care in after pediatric hematopoietic stem cell transplantation”.

The study respected the ethical principles regarding human research contained in both the Helsinki Declaration^13^, at the global level, and in Resolution 466/12, at the national level. It was approved by the research ethics committee responsible under Approval Certificate number 19772813.8.0000.0102. Therefore, the study respected the formal requirements contained in the national and international standards regulating research involving human beings.

## Results

The proposed theory was composed of 19 concepts, subdivided into the five components mentioned above that composed the social process interpreted in this phenomenon and were interpreted in the light of the symbolic interactionism. Three concepts composed the causes, that is, they represent the reason why the phenomenon happens; three address the context, which expose the meaning acquired by things to guide actions, feelings and related interactions; four brought together the intervening conditions, which are the contingencies that modify events, feelings or meanings; five approached the strategies, which give light to the understandings and responses to the occurrences made to oneself and to others; and finally, four represented the consequences, which are the results of the actions carried out or idealized.

These 19 concepts were supported by some propositions, being proposition considered the linguistic expression coming from a mental formulation. It is, therefore, the expression of actions coined in the thought, originating from the interpretation of the data. Each proposition was elaborated from the organization and relationship between categories, subcategories and codes.

From this social phenomenon represented by the proposed theory, one of the concepts that compose the strategies developed in the experience of family home care after pediatric HSCT stood out: the care provided by family caregivers. For this reason, we made this cut on the study for this concept. Being a strategy within the social phenomenon, it represents the actions that the participants develop in the face of the meanings attributed to this experience. It is emphasized that in the decision making for action in this context, the subject considers the interferences of themselves and their social environment.

The interpretation of the concept “Caring for the child” is presented, which is explained through eight propositions, which can be synthesized in the following care: administering medications; attention to cleaning issues; care with water and food intake; care with the body; experiencing protective isolation; addressing the child’s need for emotional support; addressing the child’s self-care; and facing treatment complications.

Regarding “administering medications”, the caregiver recognizes the importance of the medications to the patient and therefore assumes the responsibility of administering these medications correctly, even in face of the quantity and variety thereof. For this, although having difficulties with schedules and nomenclatures, they seek to interact with the child to facilitate acceptance, which can be identified in the participant’s speeches: *What got in my way was just the medication, which is in great quantity. So, I got a little worried, you know?! (...) And there are medications to be given until eleven pm, and then the concern to get up early; it’s tough. (...) Because I have to take care of him, and be careful not to forget the medication, to do it at the right time* (SG1P1). *So I started creating tactics like a given medicine I had to give with grape juice, the other she could take with milk, I would give with the chocolate milk, if it was a tablet I would smash, dissolve for her* (SG2P6). *But it was very sacred to me the part of the remedies; it was sacred, because they help a lot, right?!* (SG3P4).

Regarding “cleaning issues”, it is clear that the cleanliness and hygiene for the HSCT patient are differentiated from that of other people, which generates concern on the part of the caregiver with the cleaning of the environment, the preparation of food, the hygiene of the child and the people who get in touch with them or the environment they attend. There are no difficulties to perform these care, although caregivers recognize unfavorable aspects, such as the time taken, the greater use of materials and the change of behavior of the caregiver, who can exceed in this type of care: *I have to boil water, sterilize every time she eats something, I have to sterilize, I have to iron clothes. Oh God, I do not like ironing clothes, inside and out, so we have something to do the whole day; it’s a hard work* (SG1P7). *I think that part of care, anyway, was the simplest part. (...) We had everything very clean. One day, she sat down, she went to the bathroom, and I had not rubbed alcohol in the toilet; I did not sleep at night in fear... It was clean, but I had not rubbed alcohol and then I got that boggle all night: My God, I did not rub alcohol, I did not rub alcohol, what now, what now, what now?!* (SG2P7).

Taking into account the “care with water and food intake”, caregivers describe that food is another differentiated care for the transplanted children. Keeping a child fed and hydrated is one of the main concerns of the caregiver, who ends up adapting the diet of the other members of the household to the needs of the patient. This type of care represents the greatest difficulty for the caregiver, since the acceptance of food by children under treatment is not usually adequate, but this acceptance improves over time: *He did not want to eat anything because he was pretty sick of the drugs, cyclosporin, those things all. (...) Only boiled water, food cooked, only cooked, only cooked fruits in the beginning. (...) And sometimes it was difficult to do what he wanted to eat, sometimes what he wanted he could not, sometimes what he could he did not want to eat (...) I tried different things for him to eat* (SG3P7). *Because he had to take that boiled water, but then we got used to it. It had to boil; I used to boil a lot of water, so his siblings were already taking the same water. Taking good care of his health, I already took care of the others. As for food, he has never been sick like this. Oh, I could not wait for the 100 days to pass (...) We stay in that expectation, right, there are many, many things he could not. So, it was always the same thing: Can I already eat this? He used to eat until he could stand that anymore, right?!* (SG4P6).

With regard to “care with the body”, the care given to the child involves protection against the sun, the use of a mask, manipulation of the catheter and maintenance of good physical appearance, so the family routine is also modified: *Here, at home, we all used to wear mask, because I had pity of her, only her using mask. But when she was outside, she was always with the mask and umbrella, because she could not be exposed to the sun; (...) I took care of the catheter; (...) we are now taking care of her teeth* (SG3P4). *We have to be careful with the sun (...) so when you get out of the car you have to cover everything around so she is not exposed to the sun. Umbrella, if I go with her from here to the car and it is sunny, I have to open the umbrella* (SG4P8).

As for “experiencing protective isolation,” it is said that it imposes restrictions on where to go and with whom to be in touch. For this reason, the caregiver prepares people in advance, informs about the restrictions imposed and, with measures like these, adapts to the new routine. Although the caregiver recognizes the importance of isolation, it causes difficulties, such as lack of companionship, not being able to keep pets and complications to solve problems in places where the child cannot be together; potentiated situations when there is isolation by colonization by microorganisms, in addition to protective social isolation: *Actually, for me, now this is normal, (...) We could not have contact with people, could not go to places* (SG1P9). *We cannot get out, not even to the market. (...) In the first days, everyone wanted to come and visit, hence I said: he can’t, he cannot receive visits. (...) I had to say he could not receive visits, I could not be ashamed. Some people got upset, angry ... I am taking care of my child, you know ... the important thing is for him to be well* (SG3P6).

With regard to “addressing the child’s need for emotional support,” we noticed that the child is fragile and therefore emotional support is an important care. In this sense, the caregiver tries to make the environment more welcoming, seeks to be positive and confident before the child, makes company, discourages comparisons with other children who also experience or had experienced the HSCT, seeks not to victimize the patient and slow down bad feelings, motivating them to go on: *I tell him, have a little patience, boy. (...) Sometimes I talk to him, I talk and explain things to him, so he does not keep putting things in his head* (SG1P4)*. So, I was always trying to motivate, encourage her; she is getting better and better, she is improving right ?! (...) Taking care of her self-esteem all the time, you know?!* (SG3P2).

Regarding “the child’s self-care”, it is proposed that the caregiver, in addition to provide care, should also address the child’s self-care. They recognize that this is important, realize the child’s efforts to care for themselves, encourage them to take care of themselves, and even feel relief when there is a collaboration for care: *I say, daughter, today it is up to you, we have already done what we should. In our days, we did it. Today it depends much more on you than on us. Today she takes care, what it’s up to her, she takes care* (SG3P3). *I also gave responsibility to her. That hour, that medicine; she would wake up prepared. It became a routine, until she got into the routine* (SG2P6). However, in some cases, perhaps because of codependency or fear, the caregiver ends up inhibiting the child from taking an active role in their own care: *We would not let him take risks, so we would put him sit down, hand everything over, did not let him do nothing* (SG3P5).

Finally, “facing complications from the treatment” implies that clinical complications are, to some extent, expected in the HSCT process, which, in some cases, leads the caregiver to understand the patient’s situation as adequate even when there are signs or symptoms of a possible complication. This also generates a constant state of alert or fear that these situations may occur, which is why the caregiver starts having attitudes that aim to previously identify these complications. There is a greater difficulty of caring concerning a clinical complication or sequel: *Not that she had any problems related to transplantation, a sequel, but it was just nauseas, she could not feed herself, she was weakened she became thin* (SG3P1). *When he had a headache I was already fighting for them (health professionals) to do a CT scan. And I check his body from head to toe every day. Nail pain, anything different I already get stressed, I already ask* (SG1P6). However, the caregiver seeks to remain calm, because they realize that they should live the best way possible when one of these types of change occur. It is quite common, at such times, to attribute to God the non-occurrence of these changes or their improvement: *Because it’s no good for me to be desperate, you know, crying. If it did, she was already cured a long time ago, (...) I try to face the treatment in a good way, to think that everything is going to work out, that everything will end well* (SG4P8). *She went through all the difficulties, but it was thank God.* (SG2P7).

## Discussion

There has been, especially at an international level, a growing change in the care of post-HSTC patients from the hospital to the outpatient setting, which results in a greater and more rapid accountability of family caregivers in the face of direct patient care that is needed^1^ ^,^ ^5^ ^,^ ^14^. Especially when the patient is a child, the care that these caregivers assume requires attention and dedication in performing various tasks, covering instrumental, emotional and social issues of this child.

The roles and responsibilities of the family caregiver are therefore multiple^4^ ^,^ ^7^ ^,^ ^15^ ^)^ and require time, since post-HSCT patients have activities of care throughout the day and in a daily basis^7^. Although the literature does not specifically consider which care they perform, it is possible to note more emphasis on the instrumental issues^7^. It is also argued that the emotional aspect would be the most influential for the posttraumatic growth of the adult HSCT patient, but it was still concluded that the instrumental care assumed this direct relationship^15^.

This study reinforces the importance of instrumental care without, however, minimizing the social and emotional care, since subjective care can sometimes remain invisible even to those who provide or receive it^7^. Several studies mention care with medications and with cleaning and disinfection^7^ ^-^ ^16^. In this study, however, these and other issues are described when it comes to instrumental care, such as acquisition, organization and administration of medications; cleaning and disinfection of the environment and of various materials and utensils; hygiene of the child, of themselves and even of the people with whom they have contact; acquisition, handling, preparation, disposal and adequacy of the quantity and quality of food and liquids; precaution with sun exposure; imposition of mask use; handling and protection of central and/or peripheral catheters; maintenance of personal appearence; and prevention and control of complications or sequelae from treatment.

It is easy to see that child care, in this context, has different characteristics when compared to healthy children or in other situations of illness. After hospital discharge, the patient needs to assume a new lifestyle, different from what they had previously lived^7^. In order to maintain the quality of life, it is necessary to acquire new knowledge, skills and attention to the therapeutic impositions of HSCT^6^ ^-^ ^7^, which also requires the caregiver to adapt to the necessary care. These different characteristics are highlighted in this study in association with cleaning and food and water intake, which need to be specific, repetitive and of excellence.

The need to deal with the complications or sequelae of the treatment is a type of care that requires too much from the caregiver’s physical and emotional aspects. They are often already aware of the possibility of these events, which makes it easier to accept and act when they happen^1^. However, this caregiver lives with the fear that such complications they lead to relapse of the disease, or that they are not prepared to provide care when they occur^16^. Therefore, unpredictability coexists in the post-HSCT period due to the lack of guidelines on what should be expected, but the more predictable a situation is, the lesser the negative impact for the caregiver, who may assume different roles^4^.

In addition to the instrumental care, subjective, emotional and social care were also identified, such as coping with social isolation; adequacy of the family routine to the care needs of the sick child; meeting the child’s emotional needs; and promoting self-care in order to boost the child’s autonomy.

The issues related to social isolation and self-care are complex for the caregiver, since they are not prepared to deal with the limits and barriers that such care imposes^16^. Social isolation is important for HSCT because it prevents possible cross-contamination, but it is a tough reality to be experienced by both the patient and the caregiver^16^ ^-^ ^17^.

On the other hand, the encouragement, strengthening and adherence to self-care are actions that strongly relies on the family caregiver and enable the patient to continue treatment, as they encourage the acceptance of the parient’s physical, eating and social restrictions, among others^7^. However, it is difficult for the caregiver to know how much he or she needs to assign self-care to the patient, and how much this may or may not be beneficial to treatment^16^.

The importance of caring for the emotional needs of patients submitted to HSCT is crucial, since psychosocial problems lead to changes in the child’s health-related quality of life over time^18^. Thus, the relevance of the previous preparation of the family caregiver to deal with the emotional issues of the patient is emphasized^4^, especially when considering that the emotional support of the family caregiver is a facilitator for the patient to overcome the difficulties of the treatment^17^.

This study has as a limitation the fact that it was carried out in a single hematopoietic stem cell transplantation center, which, in part, includes the experience at the national level, since it serves patients of all the regions of the country; however, it does not present the scope of the caregivers’ experience in an international scope. Thus, another study to be carried out in a different nationality is suggested, so that the realities of the investigated phenomenon can be compared.

The concern with the family caregiver in the scope of HSCT is a growing reality. We believe that this work has added to the construction of this knowledge, since, in describing what types of care these subjects perform, it allows the understanding of their real needs of preparation and orientation for action. It should be emphasized that the caregiver’s preparation for home care is an action of the health team, especially the nurse, which has an expanded view of health care that goes beyond the hospital scope, with an impact on the results of transplantation, development of complications and on health care.

## Conclusion

The objective of this study was reached as it revealed the types of care carried out by family caregivers of children submitted to HSCT. Such care is multifaceted, encompassing instrumental, emotional and social aspects and, because of this, they require extensive preparation from these subjects, which covers not only the transmission of technical guidance, but also the strengthening and direction for action in the different aspects of care that will be carried out.

There is no way to minimize the role of health professionals in this situation; it is up to them to know the care taken by the family caregiver and from them develop an action plan directed to the improvement of this individual as caregiver and human being, with feelings, expectations, doubts and needs to act with the child, with their social network and with themselves.

Therefore, we consider that the results of this study represent an advance for the strengthening and organization of nursing actions in hematopoietic stem cell transplantation services, since they train these professionals to recognize the caregiver as a collaborator and to better develop their professional role so as to provide to the caregiver and the patient a care that meets their demands, that is more effective, without suffering, and providing greater understanding to the caregiver about the actions they perform and the decisions they will need to make.

This study presents the limitation of involving only pediatric caregivers; therefore, similar research should be conducted considering the caregiver of the adult patient so that they can understand the similarities and divergences between the informal care of children and adults in the period after hematopoietic stem cell transplantation.

## References

[1] Terrin N, Rodday AM, Tighiouart H, Chang G, Parsons SK (2013). Parental emotional functioning declines with occurrence of clinical complications in pediatric hematopoietic stem cell transplant. Support Care Cancer.

[2] Niederwieser D, Baldomero H, Szer J, Gratwohl MAM, Aljurf M, Atsuta Y (2016). Hematopoietic stem cell transplantation activity worldwide in 2012 and a SWOT analysis of the Worldwide Network for Blood and Marrow Transplantation Group including the global survey. Bone Marrow Transplant.

[3] Norberg AL, Forinder U (2016). Different aspects of psychological Ill health in a National Sample of Swedish Parents after successful paediatric stem cell transplantation. Pediatr Blood Cancer.

[4] Von AD, Spath M, Nielsen A, Fife B (2015). The caregiver’s role across the bone marrow transplantation trajectory. Cancer Nurs.

[5] Wulff-Burchfield EM, Jagasia M, Savani BN (2013). Long-term follow-up of informal caregivers after allo-SCT: a systematic review. Bone Marrow Transplant.

[6] Nascimento JD, Lacerda MR, Girardon-Perlini NMO, Camargo TB, Gomes IM, Zatoni DCP (2016). The experience of family care in transitional support houses. Rev Bras Enferm.

[7] Lao IF, Arranz AS, Berenguer MDP (2013). Perception of care by family caregiver. Index Enferm.

[8] Gomes IM, Hermann AP, Wolff LDG, Peres AM, Lacerda MR (2015). Grounded theory in nursing: integrative review. Rev Enferm UFPE on line.

[9] Oliver C (2012). The relationship between symbolic interactionism and interpretive description. Qual Health Res.

[10] Gomes IM Lacerda MR, Rodrigues JAP Nascimento JD, Camargo TB (2016). Use of external interviewers in qualitative research: action plan. Enferm Glob.

[11] Hutchison AJ, Johnston LH, Breckon JD (2010). Using QSR-NVivo to facilitate the development of a grounded theory project: an account of a worked example. Int J Soc Res Methodol.

[12] Santos JLG, Erdmann AL, Sousa FGM, Lanzoni GMM, Melo ALSF, Leite JL (2016). Methodological perspectives in the use of grounded theory in nursing and health research. Esc Anna Nery Rev Enferm.

[13] World Medical Association (1997). Declaration of HelsinkiRecommendations Guiding Physicians in Biomedical Research Involving Human Subjects. JAMA.

[14] Poloméni A, Lapusan S, Bompoint C, Rubio MT, Mohty M (2016). The impact of allogeneic-hematopoietic stem cell transplantation on patients’ and close relatives’ quality of life and relationships. Eur J Oncol Nurs.

[15] Nenova M, DuHamel K, Zemon V, Rini C, Redd W H (2013). Posttraumatic growth, social support, and social constraint in hematopoietic stem cell transplant survivors. Psychooncology.

[16] Jim HS, Quinn GP, Barata A, Cases M, Cessna J, Gonzalez B (2014). Caregivers’ quality of life after blood and marrow transplantation: a qualitative study. Bone Marrow Transplant.

[17] Manookian A, Nasrabadi AN, Asadi M (2014). Children’s lived experiences of hematopoietic stem cell transplantation. Nurs Health Sci.

[18] Loiselle KA, Rausch JR, Bidwell S, Drake S, Davies SM, Pai ALH (2016). Predictors of health related quality of life over time among pediatric hematopoietic stem cell transplant recipients. Pediatr Blood Cancer.

